# Inter- and Intraspecific Variation in Dorsal Pigmentation Patterns of Skates (Rajidae) from the Cantabrian Sea: Evidence of High Variability in *Raja clavata* and Implications for Species Identification

**DOI:** 10.3390/biology15191698

**Published:** 2026-09-23

**Authors:** Clara Piedrafita-Zubillaga, Daniel A. Gómez-Lobo, Isabel Gómez-Álvarez, Johanne Wauters, Pablo de la Calva-Castiñeira, Trinidad Pérez, Ángel Muñoz, Yaisel J. Borrell, Laura Miralles

**Affiliations:** 1 Department of Functional Biology, Genetics, University of Oviedo, 33006 Oviedo, Asturias, Spain; uo309264@uniovi.es (C.P.-Z.); gomezdaniel@uniovi.es (D.A.G.-L.); isabelgomal11@gmail.com (I.G.-Á.); johanne.wauters@imbrsea.eu (J.W.); uo291996@uniovi.es (P.d.l.C.-C.); pereztrinidad@uniovi.es (T.P.); borrellyaisel@uniovi.es (Y.J.B.); 2 Nueva Rula de Avilés, 33400 Avilés, Asturias, Spain; amunoz@ruladeaviles.es

**Keywords:** COI barcoding, skate conservation, morphological conservatism, phenotypic variation, polychromatism

## Abstract

Accurate species identification is essential to assess population trends and implement effective conservation measures. Many rajid species have life-history characteristics that increase their vulnerability to fishing mortality, while their conservation status differs among species. However, species identification within the family Rajidae is hampered by morphological conservatism and intraspecific phenotypic variation, which frequently lead to misidentification in fisheries surveys and seafood markets. Here, we combine COI DNA barcoding with a detailed photographic assessment of dorsal pigmentation patterns in 484 skate specimens collected from a fish wholesaler market in the Cantabrian Sea. Seven species were genetically identified as *Raja clavata*, *Raja montagui*, *Raja microocellata*, *Raja undulata*, *Raja brachyura*, *Leucoraja naevus*, and *Leucoraja circularis.* Substantial overlap in pigmentation patterns was detected among genetically distinct species, while *R. clavata* exhibited nine distinct morphotypes, five of which have not been previously described. We further investigated whether *R. maderensis* individuals display dorsal patterns distinct from those of *R. clavata*. Notably, a dark-crossbar pattern, previously unreported in *R. montagui*, was recorded in this species. Moreover, an unusually extreme case of hypomelanosis in *R. microocellata* is also discussed. Together, these findings demonstrate that dorsal pigmentation alone in processed individuals is insufficient for reliable species discrimination in skates and highlights the need to integrate molecular tools into fisheries monitoring and biodiversity assessments.

## Simple Summary

Skates are cartilaginous fishes considered among the most vulnerable marine vertebrates to extinction. Accurate species identification is therefore essential for effective conservation and fisheries management. However, identification remains challenging due to the high degree of morphological conservatism within the group, which results in highly similar external characteristics among species. This challenge is further exacerbated in fish markets, where specimens are frequently landed and commercialized in processed form (e.g., with the head and tail removed), leaving dorsal pigmentation patterns as one of the few morphological features available for identification. In this study, we combined genetic analysis of 484 skate specimens from a fish wholesaler market in the Cantabrian Sea with a detailed photographic analysis of their dorsal patterns. The objective of this work was to analyze the dorsal patterns of skate specimens landed at the fish market and assess their potential for species identification. Our results revealed previously unreported dorsal patterns in *Raja clavata*, *Raja montagui* and *Raja microocellata*. Together with a discussion of how these patterns may have evolved within the family, these findings show that dorsal pattern alone is not reliable for accurate identification and highlight the need to combine genetic tools with fisheries monitoring to support their conservation.

## 1. Introduction

Skates of the family Rajidae comprise a highly diverse group of approximately 160 species [1]. Despite their diversity, many species are considered highly vulnerable to extinction due to their life-history traits, including slow growth, late sexual maturity, and low fecundity, which are characteristic of K-selected organisms [2,3,4]. These biological traits, combined with intense fishing pressure in commercial fisheries and other threats, have contributed to the global decline of many elasmobranch populations [5,6]. Accurate species identification within Rajidae is often challenging because species of this group share highly similar morphological characteristics, known as morphological conservatism [7]. In addition, several skates exhibit substantial phenotypic variation within species while maintaining this morphological conservatism among closely related taxa, further complicating visual identification [8,9]. Such variability has important implications for fisheries management, biodiversity assessments, and conservation, as misidentification can affect landing statistics, monitoring programs, and traceability in seafood markets.

Recent studies have highlighted the extent of this problem. For instance, in the Cantabrian Sea, a previous study reported that 26% of skate products traded were mislabelled [10], illustrating the difficulty of distinguishing certain species based solely on morphology, which may compromise conservation and effective fisheries management of skates and elasmobranchs in general. Similarly, Refs. [11,12] discussed cases of atypically coloured individuals of *Raja clavata* (Linnaeus, 1758), raising questions about historical records of other species, such as *Raja asterias* (Delaroche, 1809), *Raja montagui* (Fowler, 1910), and *Raja brachyura* (Lafont, 1871), and suggesting that past misidentifications may have occurred. Indeed, *R. clavata* is known to exhibit considerable variation in colour, thorn patterns and texture [13,14,15], which may complicate its morphological separation from closely related species.

In relation to this, *Raja maderensis* (Lowe, 1838) was initially described as a distinct species from *R. clavata*, differentiated primarily by colouration characters, such as a constant dorsal pattern of dense light spots forming transverse bands and well-defined dark ventral markings on the disc margins, pectoral girdle, snout tip, and tail [16,17]. However, these diagnostic characters appear rather broad, given that *R. clavata* itself exhibits extreme dorsal colour variability, including dark spots, blotches and cloudy patterning [16,17], which could easily overlap with the pattern described for *R. maderensis*. The ventral dark markings proposed as distinctive for *R. maderensis* are described as being present only occasionally in some individuals [17], further undermining their diagnostic value. Subsequent genetic analyses failed to recover any distinction between the two taxa, and the taxonomic status of *R. maderensis* remains debated, with molecular evidence failing to support clear differentiation from *R. clavata* in some studies [18]. This case illustrates the difficulties inherent in using pattern and morphological characters alone to delimit skate species, particularly in highly variable taxa such as *R. clavata*.

These challenges are not exclusive to *R. clavata*. The nearly identical dorsal patterning of *R. brachyura* and *R. montagui* -often distinguished by the extent of black dots along the wing margin [7]-, in addition to the phenotypic intraspecific variation in dorsal pigmentation shown in *R. clavata*, can lead to misidentification based on morphology [7,9,10,11,12]. This pattern similarity not only causes misidentification between these species (*R. clavata*, *R. brachyura* and *R. montagui*) but also between these species and *R. asterias* and *Raja polystigma* (Regan, 1923) [10,12,15].

Such difficulties in morphological delimitation inevitably raise the question of whether some of this variation might reflect cryptic diversity. This possibility has already been discussed in *Raja miraletus* (Linnaeus, 1758) [19] due to the presence of identical morphological traits despite mitochondrial variation defining different lineages. Genetic analyses, specifically DNA barcoding, can provide an independent molecular line of evidence for species identification [20,21], which is often overlooked in morphological studies. Although mitochondrial COI is widely used for species identification and DNA barcoding in elasmobranchs, it has limitations since reliance on a single mitochondrial marker may not always reflect the complete evolutionary history of closely related species. In particular, and also theoretically, mitochondrial similarity may result from processes such as introgression following hybridization or incomplete lineage sorting, potentially obscuring species boundaries and relationships. Thus, the aim of this work is to document the dorsal pigmentation diversity of skates from the Cantabrian Sea for the first time in this area through a combined morphological and molecular approach, using DNA barcoding of the COI gene to provide reliable species identification.

## 2. Materials and Methods

### 2.1. Sampling

A total of 484 skate specimens (Rajidae) were collected from November 2024 to April 2025 (Supplementary Table S1) from one of the most important wholesale fish markets of the Cantabrian Sea. Boats landing at this market primarily fished in Asturias, followed by Galicia, the Basque Country, and Cantabria, in decreasing order of importance. In detail, all samples from this study were caught by artisanal fisheries in waters off the coast of the Asturias region except for one sample originating from the region of Cantabria. Approximately 63.4% of samples came from central Asturias, 11.6% from the western coast of Asturias towards Galicia, and 7% from the eastern part of Asturias. The remaining samples were fished in various combinations of these defined areas. Vessel information (name, origin, type, fishing ground and date) corresponding to all sampled individuals was recorded.

In the primary fish market, some individuals were sold whole, while others were partially processed with some sections removed, such as the head or tail, which contain characters traditionally used for morphological identification. Skates were sorted by fishermen by species and arranged in boxes prior to sale. Boxes were labelled according to vessel and fishing date, allowing specimens to be traced to individual vessels and fishing events. Every individual within a box was sampled for DNA extraction by cutting a small tissue sample (2–5 cm^2^) and preserved in 96% ethanol at 4 °C. A detailed visual assessment of dorsal pattern diversity was recorded, and all specimens were photographed to document the full range of colouration patterns present.

### 2.2. Morphological Classification

Morphological classification was performed independently of molecular identification, with observers blinded to the genetic results to ensure that morphological assignments were not influenced by molecular data.

Characterisation traits were established by consensus after carefully reviewing photographs of all individuals and were selected as the least subjective traits for distinguishing morphotypes. These include (i) the size and shape of black dots, assessed visually along a gradient from fine, circular stippling to larger, elongated and denser markings; (ii) the presence, distribution, and relative extent of white markings, including whether they occurred on the wings or were restricted to the central disc; (iii) the presence or absence of ocelli; and (iv) background body colour, scored visually as dark or light. A character matrix defining the nine morphotypes of *Raja clavata* can be consulted in Supplementary Figure S1. Morphotype classification was performed blind to molecular species identity and was independently repeated three times on separate days to assess consistency among the authors and the main classifier. Pigmentation photographs were obtained under field conditions (opportunistic sampling in a commercial landing setting), using personal smartphone devices operating in automatic mode for autofocus, automatic white balance, and automatic exposure; with consumer-grade cameras of ≥12 MP resolution. No manual control was applied over ISO, shutter speed, or colour temperature. Distance, angle, and ambient lighting were not standardised across specimens, and no colour reference cards were included in the photographs, since time was a pressure limitation with individual manipulation minimised. For figure presentation, the background was removed from the images to improve specimen visibility, without modifying the pigmentation patterns or other morphological features.

### 2.3. Genetic Identification

DNA extraction from all specimens was carried out using a Chelex-based protocol [22]. Species identification relied on DNA barcoding of a 704 bp fragment of the mitochondrial Cytochrome Oxidase subunit I (COI) gene, amplified with FishF2 and FishR2 primers following the protocol, reaction volumes, thermal cycling conditions, and primer concentrations described in [23]. Sequencing was performed by Macrogen Inc. (Madrid, Spain) using a 3730XL DNA Analyzer (Applied Biosystems^TM^, Foster City, California, USA). Sequence processing and alignment were completed in Geneious Prime 2025.0.3. Taxonomic identification was inferred through BLAST searches against GenBank (http://blast.ncbi.nlm.nih.gov/Blast.cgi, accessed on 1 September 2026) and BOLD systems (www.boldsystems.org, accessed on 1 September 2026) databases, applying a ≥98% identity threshold [24]. Based on previous studies [18], it could be possible that for the species *R. maderensis*, BLAST similarity values alone were not considered sufficient for species-level identification, and previously published taxonomic and genetic information would also be considered, if necessary.

Each individual chromatogram was manually checked. Species identification in BOLD was performed using the Species Level Barcode Identification tool. In GenBank, each sequence was compared with the reference sequence showing the highest identity percentage and query coverage. All detected mismatches in our dataset with the best-matched reference sequences from the database were verified in Geneious 2025.0.3. Mutations were accepted only after revising the translation into amino acids to exclude stop codons, unexpected insertions or deletions, and sequencing artifacts, and only when the quality Phred scores were high (QV > 20). A reference genome for each of the species was used in Genious 2025.0.3 to obtain the COI primer binding sites. Thus, amplified fragments of approximately 704 bp were trimmed according to the primer binding sites, giving a final amplified region of 619 bp for all species.

A Maximum Likelihood analysis was performed for the COI gene to visually map the genetic distances between the identified morphotypes. The ML analysis consisted of bootstrapping 1000 initial trees following a GTR + GAMMA evolutionary model implemented in RAxML GUI 2.0.

## 3. Results

COI sequencing was successful for 483 specimens, yielding a final dataset of sequences 619 bp in length. COI sequencing of one individual failed, yielding a low-quality sequence. Seven species were identified: *R. clavata* (*n* = 287; 59.4%), *R. montagui* (*n* = 72; 14.9%), *Raja microocellata* (Montagu, 1818) (*n* = 45; 9.3%), *Raja undulata* (Lacepède, 1802) (*n* = 27; 5.5%), *R. brachyura* (*n* = 18; 3.7%), *Leucoraja naevus* (Müller & Henle, 1841) (*n* = 33; 6.8%), and *Leucoraja circularis* (Couch, 1838) (*n* = 2; 0.4%). Of the 287 *R. clavata* individuals, 18 returned a top match to *R. maderensis* (6%); however, these were assigned to *R. clavata* according to the most recent genetic findings [18]. Species assignments were highly consistent between GenBank and BOLD, with all top matches showing ≥99% similarity (Supplementary Table S1).

**Figure 1:**
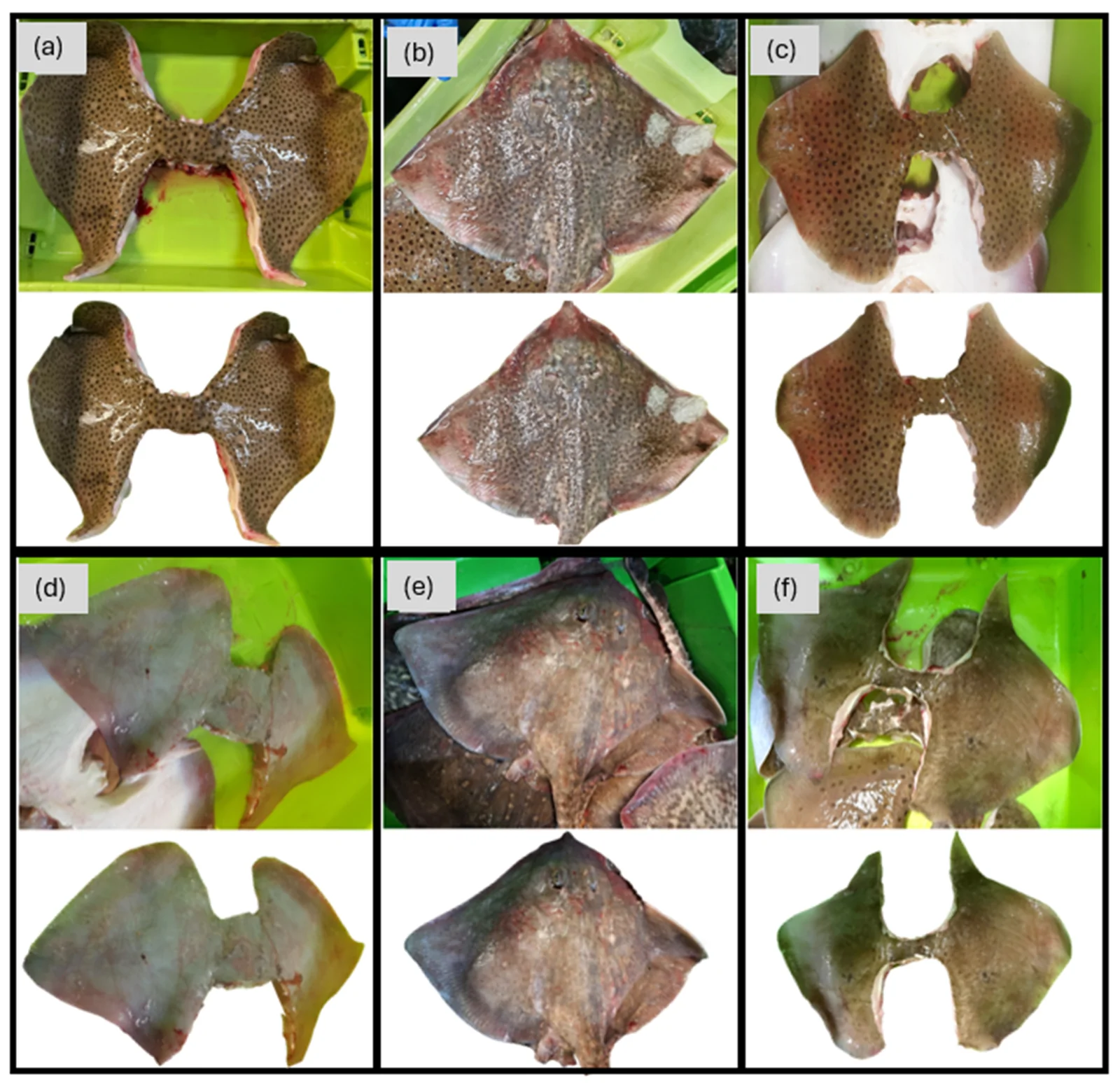
Highly similar dotted and plain patterns present across different species. Original pictures from the fish market above and below simplified individuals without background. (**a**) Dotted pattern in *R. brachyura*. (**b**) Dotted pattern in *R. clavata*. (**c**) Dotted pattern in *R. montagui*. (**d**) Plain pattern in *R. microocellata*. (**e**) Plain pattern in *R. clavata.* (**f**) Plain pattern in *R. montagui*.

High phenotypic variation among skate species was evident during sampling (Figure 1). Individuals sharing a spotted dorsal pattern were recorded across *R. clavata*, *R. brachyura*, and *R. montagui*, despite belonging to different species (Figure 1a–c). Similarly, a plain or unpatterned phenotype was also observed across multiple species, including *R. microocellata*, *R. montagui*, and *R. clavata*, the latter representing a common morphotype within this species (Figure 1d–f).

**Figure 2:**
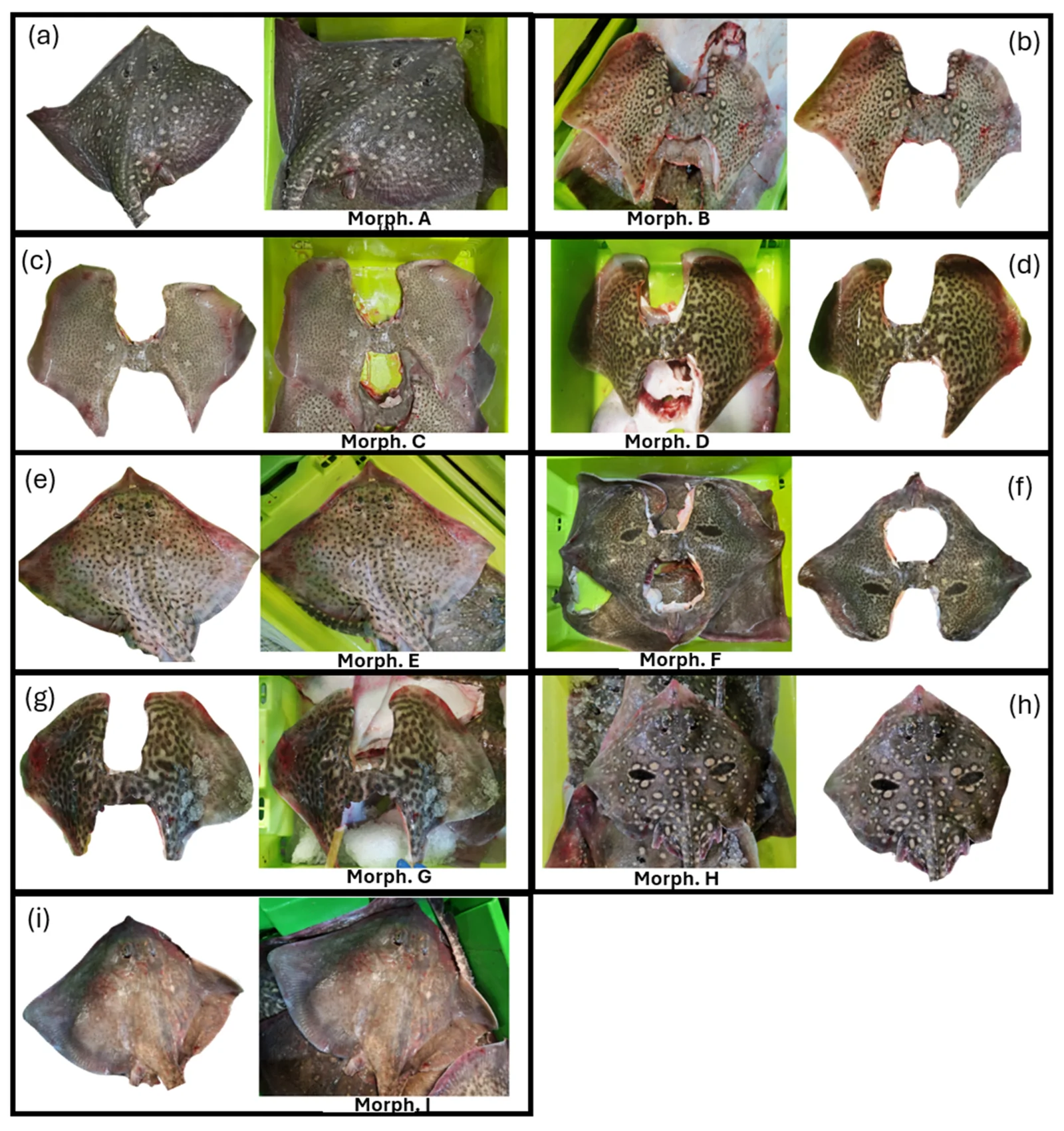
Dorsal pigmentation morphotypes identified in *R. clavata* (*n* = 241). Original pictures from the fish market were presented, and simplified individuals without background were illustrated. (**a**) Morph A—‘Starlit’. (**b**) Morph B—‘Central white-spotted’. (**c**) Morph C—‘Sandy’. (**d**) Morph D—‘Leopard’. (**e**) Morph E—‘Plain-spotted’. (**f**) Morph F—‘Ocellate central white-spotted’. (**g**) Morph G—‘Ocellate leopard’. (**h**) Morph H—‘Ocellate starlit’. (**i**) Morph I—‘Plain’.

This variability was observed between species but also within species, particularly in *R. clavata*, which exhibited distinguishable morphotypes (Figure 2), further complicating identification. A total of 287 *R. clavata* (and *R. maderensis*) individuals were examined, of which 242 (84.3%) could be assigned to one of the defined morphotypes; the remaining 46 individuals correspond to individuals showing intermediate or atypical combinations of features, or for which the available photographs did not allow a reliable classification. Those individuals were recorded separately and were not assigned to a specific morphotype (Supplementary Table S1). A total of nine morphotypes were defined as descriptive categories representing the main recurring dorsal pigmentation patterns observed in *R. clavata*. They should not be interpreted as discrete phenotypic categories, but rather as convenient descriptors within a continuous range of pigmentation variation.

The nine distinct morphotypes were identified for *R. clavata* based on dorsal pigmentation patterns (Figure 2). Morphotypes were coded with letters (A–I) and assigned a descriptive common name to facilitate identification. Morph A, ‘Starlit’ (*n* = 46; 19%); Morph B, ‘Central white-spotted’ (*n* = 69; 28.5%); Morph C, ‘Sandy’ (*n* = 38; 15.7%); Morph D, ‘Leopard’ (*n* = 24; 9.9%); Morph E, ‘Plain-spotted’ (*n* = 13; 5.4%); Morph F, ‘Ocellate central white-spotted’ (*n* = 29; 12.0%); Morph G, ‘Ocellate leopard’ (*n* = 5; 2.1%); Morph H, ‘Ocellate starlit’ (*n* = 12; 4.9%); and Morph I, ‘Plain’ (*n* = 6; 2.5%) are described in detail and summarised in Table 1 and Supplementary Figure S1.

**Figure 3:**
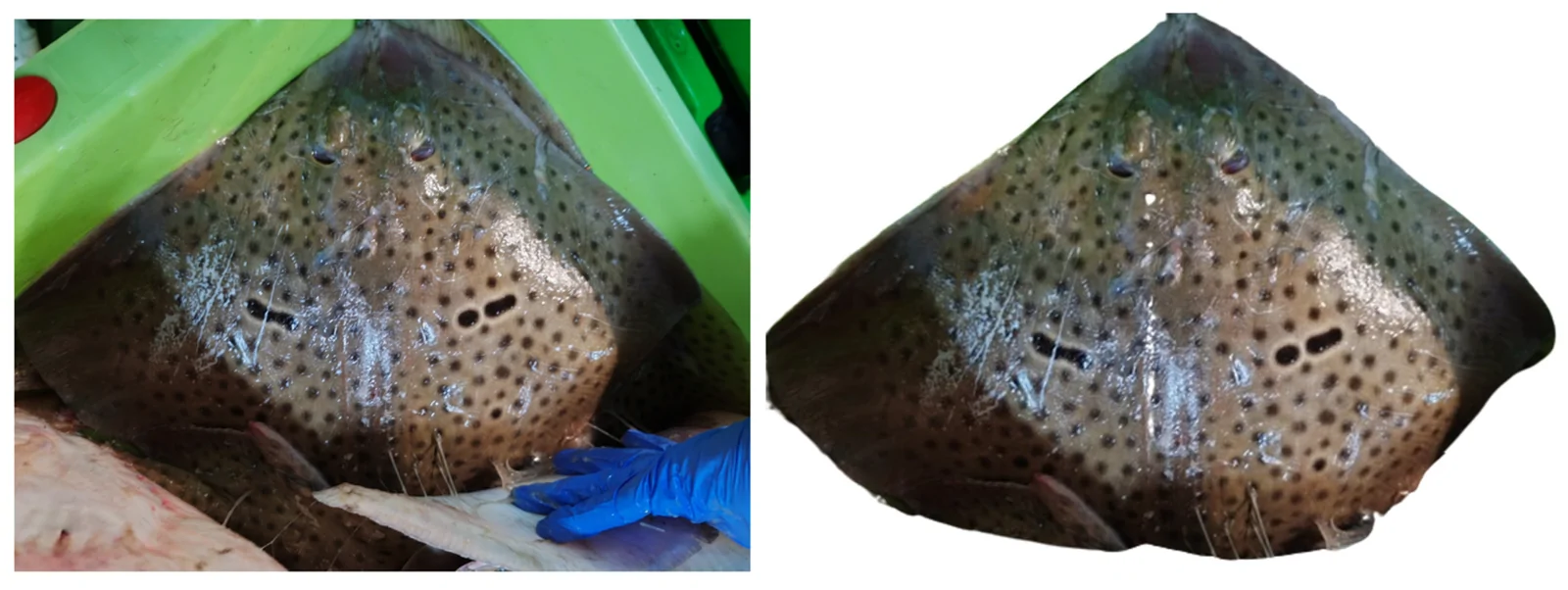
Dark-crossbar pattern in a single genetically identified *R. montagui* individual. Original picture from the fish market (**left**) and simplified individual without background (**right**).

In addition, one individual of *R. montagui* exhibited a distinct dorsal pattern featuring paired dark crossbars (Figure 3).

**Figure 4:**
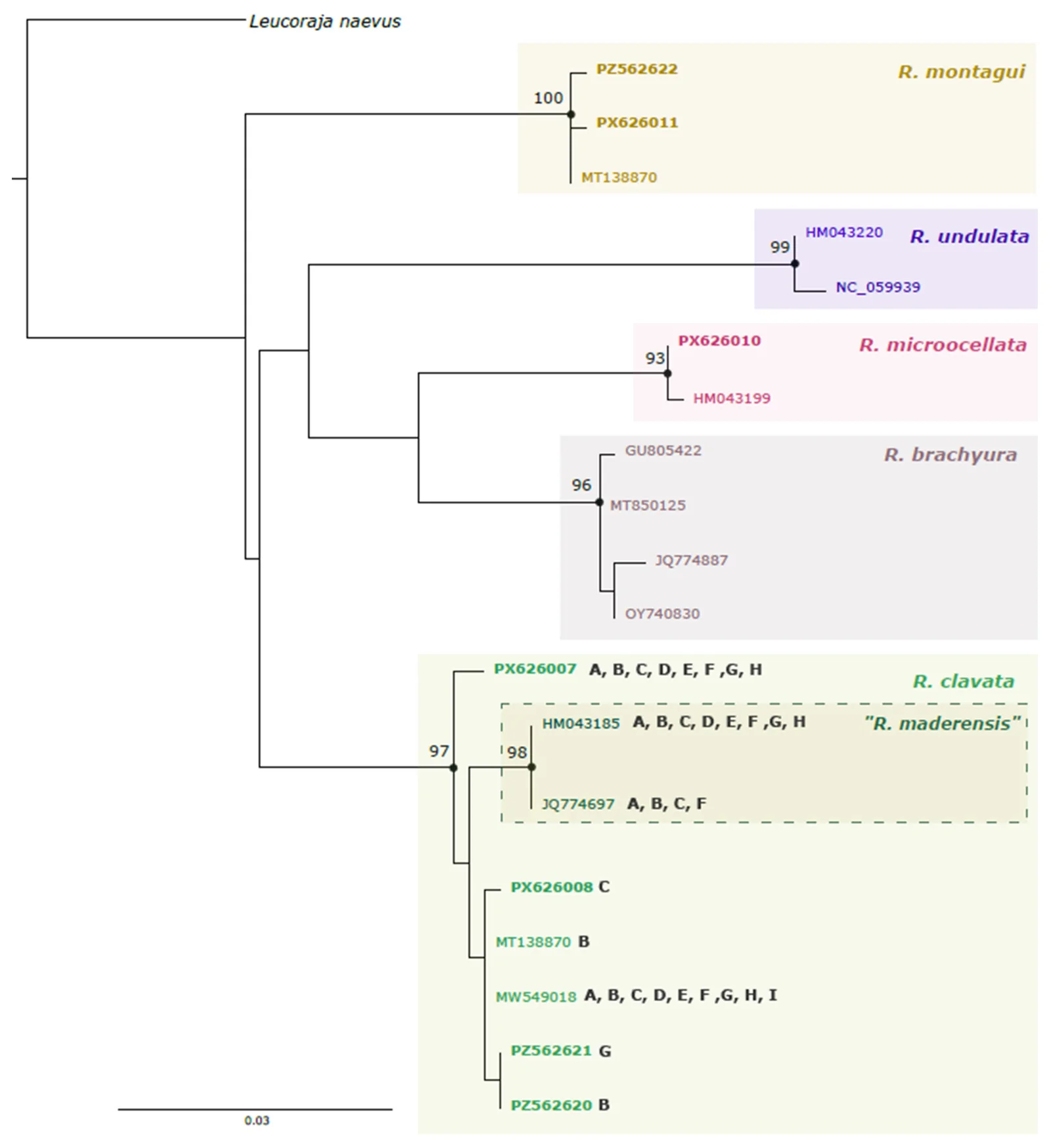
Maximum Likelihood phylogenetic tree based on the 619-bp COI region. The tree was reconstructed under a GTR + Gamma evolutionary model with 1000 bootstrap replicates. Bootstrap support values (>90%) are indicated at the corresponding nodes. Accession numbers identify the reference COI sequences included in the analysis, and letters A–I indicate the pigmentation morphotypes represented by each haplotype. Each species is represented with one colour and *R. maderensis* is enclosed with a dashed line box inside *R. clavata*. *Leucoraja naevus* was used as the outgroup. The scale bar represents nucleotide substitutions per site.

The ML phylogenetic tree (Figure 4) separates each of the Rajidae species included in our study into well-supported clades (BS > 90%). Within the *R. clavata* clade, a well-supported subclade forms, with *R. maderensis* nested within *R. clavata*. Morphotypes A–H are present in haplotype PX626007, HM043185 (*R. maderensis* haplotype), and MW549018; the latter also includes Morph I, “Plain”. The remaining haplotypes of *R. clavata* and *R. maderensis* were each represented by only one individual, except for haplotype JQ774697, which included six individuals. Thus, these haplotypes appear to be associated with only one morphotype (or four, in the case of JQ774697). The ML analysis does not show a clear correspondence between morphotypes and haplotypes; instead, it reflects the full variety of morphotypes across all clades of *R. clavata* and *R. maderensis*. The only morphotype that appears exclusive to a single haplotype is Morph I, “Plain”. However, as only six individuals presented this morph, this observation should be interpreted with caution.

## 4. Discussion

The present study reveals significant discordance between dorsal pigmentation patterns and genetic species identification in skates from the Cantabrian Sea. Considerable intraspecific variation was detected, particularly in *R. clavata*, in which several distinct morphotypes were identified, most of them reported here for the first time. A high degree of interspecific overlap in dorsal pigmentation patterns, with similar spotted and unpatterned phenotypes occurring across genetically distinct taxa, was observed. Collectively, these findings indicate that dorsal pigmentation patterns alone provide limited taxonomic resolution for the reliable identification of skate species in fisheries and fish markets. This limitation is especially relevant in Spain, where—despite regulations prohibiting the landing of processed skates (Regulation (EU) No 605/2013) within the broader fisheries control framework established by Regulation (EC) No 1224/2009, as amended by Regulation (EU) 2023/2842—specimens lacking the head and tail or consisting only of wings are frequently observed in most primary fish markets, leaving dorsal colouration and patterning as the main characteristics available for species identification.

These morphological similarities highlight a high degree of phenotypic variation, resulting in conserved external traits across skate species that make identification based solely on morphology challenging. Similar *R. clavata* patterns were reported by [11], who discussed possible misidentifications of *R. clavata* in the Sea of Marmara due to pronounced phenotypic variability. Notably, specimens B and C in [11] (visually identified as *R. clavata*) closely resemble individuals of *R. clavata* from our study, which fall within our Morph E, ‘Plain-spotted’. This morphotype, in turn, shows strong visual similarity with *R. montagui* and *R. brachyura*, also discussed in previous studies [11,12], further illustrating the extent of interspecific overlap in dorsal pigmentation patterns. In addition, specimen A in [11], which presents ocelli and is identified as *R. clavata*, is particularly relevant in this context. To date, ocelli-like markings have been described as part of the typical pigmentation variability of *R. clavata* [1,15] and reported only as a rare morphological aberration in the closely related *R. miraletus* [25]. However, they have not been previously documented in *R. montagui*. Here, we recorded rare individuals of *R. montagui* displaying dark crossbar markings (Figure 3), representing, to our knowledge, the first report in the revised scientific literature of this pigmentation trait in the species and further increasing the complexity of morphological identification in Rajidae.

Intraspecific variation was particularly high in *R. clavata*, where a wide range of pigmentation patterns was observed, including morphotypes that resemble other species. The pigmentation patterns observed in this study align with previous descriptions [15], with all the types described therein except type 7 being represented in our dataset. A correspondence can be established between most of their categories and our morphotypes (e.g., type 2 with Morph F, type 3 with Morph C, types 4 and 1 with Morph E, and type 6 with Morph A), while their type 5 likely represents an intermediate condition between our Morphs D and A, a pattern that we also observed in seven individuals (Supplementary Table S1). Some other morphotypes also present a qualitative gradient in their pattern trend. Morph A, ‘Starlit’, encompasses a continuous intramorph gradient ranging from individuals lacking fine black dots entirely, corresponding to the classic ‘Starlit’ pattern, to individuals displaying dense fine black dots forming a rosette pattern at the opposite extreme, as described by [12] (Supplementary Figure S1). Rather than representing two discrete morphotypes, this variation is here interpreted as a single continuous phenotypic gradient in which the rosette pattern constitutes the high-expression extreme of the same pigmentation character. The same gradient was observed in Morph H, ‘Ocellate starlit’, but with the additional presence of ocelli (Supplementary Figure S1). This interpretation is supported by the occurrence of intermediate individuals that cannot be unambiguously assigned to either extreme, suggesting that dot density varies gradually across the morphotype. However, since the gradient was also observed in specimens with apparently well-preserved pigmentation, a biological explanation is favoured over a preservation artefact. These limitations should also be considered; degradation and storage conditions prior to sampling cannot be entirely excluded as potential sources of variation in colour intensity. Accordingly, interpretations based on subtle differences in background colour are considered indicative rather than definitive, while the morphotype classifications are based on the broader and more readily identifiable pigmentation patterns. Beyond this general agreement, our results expand the known phenotypic diversity of the species. In addition, we identified five morphotypes that, to our knowledge, have not been previously reported in the revised literature, including Morph B (‘Central white-spotted’), the most frequently found in our sample (28% of *R. clavata* individuals); as well as Morph D (‘Leopard’), Morph G (‘Ocellated leopard’), Morph H (‘Ocellate starlit’), and Morph I (‘Plain’) (Figure 2). Morphotype frequencies should be interpreted as frequencies within our sampled commercial landings and should not be extrapolated to the population level, because factors influencing prevalence, such as fishing area, vessel, season, body size, and market sorting, may influence the composition of the sample. Together, these findings suggest that pigmentation variability in *R. clavata* may be greater than previously recognized.

In particular, the presence of these complex visual structures—specifically the paired ocelli-like markings in *R. clavata* (Morphs F, G, and H) and the anomalous dark crossbars in *R. montagui*—can be interpreted through the macroevolutionary framework recently proposed [26]. Within their methodology, conspicuous markings are defined as prominent patches or bands that diverge sharply from the species’ baseline body colouration, distinguishing them from the regular, uniform background spotting. Their model hypothesizes that complex eyespots in skates (as seen in *R. miraletus*, for example) do not emerge de novo, but rather through a stepwise pathway: moving from these unpatterned baseline states to multiple markings, reducing them to a symmetric pair, and finally undergoing geometric circularization. While our data do not capture the full range of these transitions (as we did not observe an intermediate ‘multiple-marking’ phase), the occurrence of these paired structures suggests a hypothetical possible link to this latent evolutionary potential. Interestingly, our results reveal a clear difference in the evolutionary standing of this character between the two species. In *R. clavata*, the markings appear more established and structured, exhibiting a degree of circularization across several recurrent morphotypes. In contrast, its expression in *R. montagui* remains highly sporadic and rare, appearing as an isolated anomaly (Figure 3). Although the crossbar phenotype was observed in only one individual in our dataset, similar pigmentation has been observed in several independent observations of additional *R. montagui* individuals from the Cantabrian Sea (C.R.C. unpublished observations, pers. comm.), suggesting that this phenotype may occur more broadly than indicated by our sample. However, whether this phenotype has any developmental or evolutionary relationship with ocelli remains unknown.

As a hypothesis to be further investigated in future research, this variation might reflect both phenotypic variation in dorsal pigmentation and underlying evolutionary processes. Given the relatively recent origin of *R. clavata*, dated to the late Miocene around the Messinian Salinity Crisis [27,28], the number of generations that have passed since divergence may have been insufficient for morphological differences to fully track genetic divergence across recently separated lineages. As a hypothesis, shared ancestral variation, incomplete lineage sorting, introgression, or other processes could potentially contribute to explaining the shared morphotypes of *R. clavata* (Morph E, ‘Plain-spotted’) among closely related species, such as *R. montagui* and *R. brachyura*, although genomic data would be needed to test these hypotheses.

The case of *R. maderensis* further illustrates this pattern. In accordance with [18], which found no genetic differentiation between *R. clavata* and *R. maderensis*, the individuals in our dataset that returned a top NCBI match to *R. maderensis* did not display any distinctive or exclusive morphotype, nor did they exhibit a unique pigmentation phenotype. Their dorsal patterns spanned all nine morphotypes described in this study (Supplementary Table S1), further supporting the lack of differentiation between the two taxa and reinforcing the idea that no clear morphological basis exists for their separation based on dorsal patterning alone. This is consistent with a broader pattern of morphological stasis across this clade: Ref. [28] reports that *R. clavata*, *R. maderensis,* and *Raja straeleni* (Poll, 1951) form a clade of sibling taxa in which divergence at mtDNA loci is minimal, attributed to insufficient time since separation for reciprocal monophyly to develop (a grouping that was only resolved through the ABGD delimitation method). This reflects a history in which lineage divergence has outpaced morphological differentiation.

The presence of rare and atypical phenotypes adds further complexity. Some individuals showed intermediate traits between *R. clavata* and *R. montagui*, the two most frequently landed species. Although this could be explained by the high phenotypic variation and morphological conservatism known in Rajidae [7,15], other processes such as introgression or hybridization, previously reported in elasmobranchs [29], cannot be excluded and warrant further investigation, as the available data do not allow these processes to be distinguished from ordinary intraspecific pigmentation variation. Additionally, one *R. microocellata* individual showed a plain, almost translucent phenotype, which might indicate the first record of albinism in the species (Figure 1d). Albinism has already been reported in Rajidae, including *R. clavata*, *R. brachyura* and *R. montagui* [30]. An unusually extreme case of hypomelanosis was observed in one individual of *R. microocellata*; however, insufficient diagnostic features were available to confirm this interpretation, such as eyes, as the specimen was incomplete (lacking the head and tail).

The results of this study highlight the limitations of relying solely on external morphology for skate identification and underscore the challenges posed by extensive phenotypic variation within Rajidae. These limitations should also be considered, taking into account the nature of our sampling, which was conducted on specimens obtained from commercial landings under non-standardised market conditions. For instance, standard morphometric measurements and holistic taxonomic characters could not be assessed. Future studies would therefore benefit from a standardised photographic protocol, including fixed distance and orientation, controlled illumination, and colour-calibration references, particularly for quantitative comparisons of pigmentation. Nevertheless, these methodological limitations are unlikely to account for the broad qualitative variation and substantial overlap in dorsal pigmentation patterns observed among genetically identified species, and these findings have implications beyond taxonomy. The high variability and overlap in dorsal pigmentation patterns, together with the presence of atypical and intermediate phenotypes, limit the reliability of morphology alone for species identification in primary fish markets. This can lead to errors in fisheries surveys and biodiversity assessments, with direct consequences for conservation and management. In this context, integrating genetic tools with morphological approaches is essential. Combining genetic and morphological data will not only improve taxonomic accuracy but also provide a more robust basis for fisheries management, conservation planning, and the assessment of elasmobranch biodiversity. Future studies incorporating nuclear markers, which can provide complementary and higher-resolution information than the mitochondrial COI marker employed here, would be needed to further clarify the evolutionary and ecological processes underlying the remarkable phenotypic diversity observed in these species. An effective monitoring framework should combine routine morphological identification with improved identification keys and training, periodic molecular auditing, and DNA barcoding of uncertain specimens. Molecular validation of market traceability datasets could further strengthen the reliability and management relevance of these approaches.

## 5. Conclusions

This study indicates that skates of the genus *Raja* exhibit high similarity in external pigmentation among the examined species, with genetically distinct species frequently sharing highly similar dorsal pigmentation patterns, while *R. clavata* displays remarkable intraspecific phenotypic variation in dorsal pigmentation, encompassing a much broader range of colour variation than previously recognized. By integrating DNA barcoding with detailed dorsal pattern observations, we provide a detailed characterization of dorsal pigmentation diversity in *R. clavata* from the Cantabrian Sea, including five previously undescribed morphotypes, while also documenting a previously unreported dorsal crossbar pattern in *R. montagui* and a putative hypopigmented phenotype consistent with possible hypomelanosis or albinism in *R. microocellata*. Together, these findings confirm that external pigmentation alone has limited taxonomic value for the reliable identification of Rajidae, particularly in commercial settings where diagnostic anatomical characters are often absent. At the same time, the morphotypes described here establish a practical framework for documenting pigmentation diversity in future taxonomic and fisheries studies. More broadly, the integration of molecular and morphological evidence provides a robust basis for improving species identification and traceability while opening new opportunities to investigate the evolutionary and developmental processes underlying colour pattern diversification in skates through future genomic and broader geographic studies.

## Figures and Tables

**Table 1:** Detailed description of *R. clavata* morphotypes recorded during sampling. In bold are the patterns not previously described in the literature reviewed by the authors.

Morphotype	Descriptive Name	Description	Reference	Figure
Morph A	Starlit	Dark background with white marks distributed across the entire dorsal surface, typically surrounded by a dark halo. Exhibits an intramorph gradient from individuals lacking fine black dots entirely (classic pattern) to individuals with dense fine black dots forming a rosette pattern at the opposite extreme	[12] (rosette extreme) [15] *sensu* type 6	Figure 2a; Supplementary Figure S1
**Morph B**	Central white-spotted	White marks largely restricted to the central area, usually bordered by a dark halo, with medium-sized black dots on a generally pale background	**This study**	Figure 2b
Morph C	Sandy	Similar to Morph B but with finer black dots and white marks only partially or not surrounded by a dark halo	[15] *sensu* type 3	Figure 2c
**Morph D**	Leopard	Leopard-like pattern of dark blotches	**This study**	Figure 2d
Morph E	Plain-spotted	Pale background with medium-sized black dots and no conspicuous white marks; reminiscent of *R. montagui*	[11,15] *sensu* types 1 and 4	Figure 2e
Morph F	Ocellate central white-spotted	Pattern as Morph B with addition of ocelli	[15] *sensu* type 2	Figure 2f
**Morph G**	Ocellate leopard	Pattern as Morph D with addition of ocelli	**This study**	Figure 2g
**Morph H**	Ocellate starlit	Pattern as Morph A with addition of ocelli; exhibits the same intramorph gradient as Morph A, from individuals lacking fine black dots (classic pattern) to individuals displaying a rosette pattern combined with ocelli at the opposite extreme	**This study**	Figure 2h; Supplementary Figure S1
**Morph I**	Plain	Plain dorsal surface devoid of markings	**This study**	Figure 2i

## Data Availability

The original data presented in the study are openly available in GenBank under accession numbers PX626007-11 and PZ562620-22.

## Supplementary Materials

The following supporting information can be downloaded at https://www.mdpi.com/article/10.3390/biology15191698/s1. Figure S1: Character matrix defining the nine morphotypes of *Raja clavata*. Figure S2: Fine black dot gradient in Morph A, ‘Starlit’, transitioning from absence (A); intermediate presence (B); and clear presence forming a ‘rosette pattern’ described by [12] (C). Fine black dot gradient in Morph H, ‘Ocellate starlit’, transitioning from total absence of black pigmentation besides ocelli (D); absence of fine black dots (E); and clear presence forming a ‘rosette pattern’ described by [12] plus ocelli (F). Table S1: Detailed information for sampled individuals of Rajidae.

## Abbreviations

The following abbreviations are used in this manuscript:

ABDG	Automatic Barcode Gap Discovery
ILS	Incomplete Lineage Sorting
